# A National, Palliative Care Competency Framework for Undergraduate Medical Curricula

**DOI:** 10.3390/ijerph17072396

**Published:** 2020-04-01

**Authors:** Jolien Pieters, Diana H.J.M. Dolmans, Marieke H.J. van den Beuken-van Everdingen, Franca C. Warmenhoven, Judith H. Westen, Daniëlle M.L. Verstegen

**Affiliations:** 1 Department of Educational Development and Research/School of Health Professions Education, Faculty of Health, Medicine and Life Sciences, Maastricht University, 6229 ER Maastricht, The Netherlands.f.warmenhoven@maastrichtuniversity.nl (F.C.W.); j.westen@maastrichtuniversity.nl (J.H.W.);; 2 Centre of Expertise for Palliative Care Maastricht UMC+, 6229 HX Maastricht, The Netherlands; m.vanden.beuken@mumc.nl

**Keywords:** undergraduate education, competency framework, medical students, palliative care education, Delphi

## Abstract

As nearly all doctors deal with patients requiring palliative care, it is imperative that palliative care education starts early. This study aimed to validate a national, palliative care competency framework for undergraduate medical curricula. We conducted a Delphi study with five groups of stakeholders (palliative care experts, physicians, nurses, curriculum coordinators, and junior doctors), inviting them to rate a competency list. The list was organized around six key competencies. For each competency, participants indicated the level to which students should have mastered the skill at the end of undergraduate training. Stability was reached after two rating rounds (*N* = 82 round 1, *N* = 54 round 2). The results showed high levels of agreement within and between stakeholder groups. Participants agreed that theoretical knowledge is not enough: Students must practice palliative care competencies, albeit to varying degrees. Overall, communication and personal development and well-being scored the highest: Junior doctors should be able to perform these in the workplace under close supervision. Advance care planning scored the lowest, indicating performance in a simulated setting. A wide range of stakeholders validated a palliative care competency framework for undergraduate medical curricula. This framework can be used to guide teaching about palliative care.

## 1. Introduction

Due to an increasingly ageing population and prevalence of noncommunicable diseases, the need for palliative care is expected to grow. This requires changes in government policies, but also in the professional training and education [1]. Physicians will regularly need to care for patients requiring palliative care. Providing this care can be challenging and calls for specific competencies, which can be mentally and emotionally taxing for health care professionals [2]. It is therefore important that junior doctors develop the ability to guide and support the chronically and terminally ill during their medical training [3]. Several studies, however, have demonstrated that medical students do not sufficiently receive such education and training in palliative care [4,5,6]. On the other hand, palliative care education has recently started to receive more attention, several education interventions are made, and the subject is now taught in a large number of undergraduate medical programmes at European universities [7]. Yet, the level at which palliative care is taught still differs widely between individual countries [7,8]. This study focused on the Netherlands as one of the European countries where palliative care [9], albeit marked as general care, still receives very limited attention in medical curricula. In a previous study [10], Dutch final-year medical students reported that their curricula, indeed, did not adequately cover many of the palliative care aspects they deemed important. This finding ties in with studies from different countries showing that students lack knowledge and confidence in caring for patients requiring palliative care [11,12,13,14]. 

Clearly, palliative care education should be developed and implemented in medical curricula [15]. What is not clear, however, is what contents the undergraduate curricula should exactly cover and what should be covered in postgraduate specialty training programmes and/or continuing professional development. The Pasemeco (Palliative care, Alliance, Sharing, Educational tools for MEdical student COmpetencies development) project focuses on integrating palliative care education into the eight Dutch medical curricula. Its project team has drafted a list of palliative care competencies based on two palliative care competency profiles that are suitable for the Dutch situation: An educational framework developed by VU University Medical Centre in the Netherlands [16] and the European Association for Palliative Care (EAPC) competencies described in a white paper [17]. We face-validated items within the project group, which contained different kinds of stakeholders. Whereas this educational framework describes the knowledge, skills, and attitudes regarding palliative care that a range of health care professionals should possess, the EAPC white paper describes the competencies that students should acquire according to European guidelines. Although the two profiles provide a solid basis, they are yet to be further refined, tested, and examined by various stakeholders.

The purpose of this study was to gain insight into what is expected of medical students at the end of the undergraduate curricula when they move into the role of junior doctor. To this end, we conducted a Delphi study by offering the draft competency framework to various stakeholders for validation. In a Delphi procedure, a panel of experts rate the framework in different rounds until consensus or stability in panel members’ responses is reached. This technique seemed a useful method to gather the opinions of various stakeholders with the aim to achieve consensus, especially since the same method has been used elsewhere to develop competency frameworks for palliative care education [18,19]. To obtain more detailed insight, we investigated not only what contents undergraduate curricula should cover, but also with what degree of autonomy junior doctors should be able to perform palliative care tasks upon graduation. For instance, would they need to perform these in a simulated setting or in the workplace? Additionally, in the latter case, under close supervision or with a supervisor on call? We expected different stakeholders to have equally different responses to these questions. More specifically, palliative care experts could hold the view that junior doctors should be able to perform palliative care tasks in the workplace with little supervision, whereas physicians and nurses could think otherwise. In summary, this study addressed the following question: Which palliative-care-related competencies should, according to palliative care experts, junior doctors, experienced physicians, nurses, and educators, be addressed in undergraduate medical curricula, and at which level?

## 2. Materials and Methods 

### 2.1. Panel

The study involved sending an online questionnaire to a panel of representatives from five groups of stakeholders (Table 1). The first group consisted of experts in palliative care: Physicians or nurses who had received specialist palliative care training. The second and third group consisted of physicians and nurses who were not specialized in palliative care but were involved in caring for patients requiring palliative care. We expected them to have insight into the abilities of junior doctors and the demands placed on them because they were either supervising them (physicians) or collaborating closely with them (nurses). The fourth group consisted of educators and curriculum coordinators from several medical universities in the Netherlands who could share their educational view on the topic. The final group consisted of recently graduated, junior doctors. We recruited these stakeholders from the networks of the Pasemeco project team and the Expertise Centres for Palliative Care (EPZ), which are linked to every University Medical Centre in the Netherlands, by sending them an email invitation to participate. Table 1 provides more details on the participants.

### 2.2. Instrument

The draft competency list was organised around six key competencies reflecting the tasks that newly graduated junior doctors are expected to perform. These key competencies covered the following domains: Communication, advance care planning, pain and symptom management, working in a multidisciplinary team, end-of-life care, and personal development and well-being. Each key competency covered three to ten enabling competencies, resulting in a list totaling 46 items (comprising six key competencies and 40 enabling competencies). An enabling competency of ‘communication’ (key competency), for instance, was: ‘Is able to communicate with respect and empathy with patients and relatives*’*. The panel members were asked to indicate per key and enabling competency (each item) if and to what degree the junior doctor should master this competency. More specifically, panel members were asked whether they expected junior doctors to only have theoretical knowledge of the competencies (basic or advanced), or to be able to practice them, either in a simulated setting or in the workplace (under close supervision or with a supervisor on call). With a simulated setting we mean practice outside the clinical setting in practice settings organized for education, for example, role plays, practice with simulated patients, or in a simulation center. This resulted in a six-point Likert scale, ranging from ‘Not applicable: Competency is not required’ to ‘The newly graduated junior doctor must be able to execute this task independently in the workplace with a supervisor available on call’ (see Box 1).

Box 1 **Explanation of the six-point Likert scale on which competencies were rated.**

**Meaning**
1Not applicable: A newly graduated junior doctor does not require this competency.2The newly graduated junior doctor must possess the basic knowledge, skills, and attitudes (professional behaviour) needed for this task.3The newly graduated junior doctor must be able to integrate the knowledge, skills, and attitudes (professional behaviour) needed for this task.4The newly graduated junior doctor must be able to execute this task in an educational or simulated setting (under the teacher’s supervision).5The newly graduated junior doctor must be able to execute this task in the workplace under close supervision.6The newly graduated junior doctor must be able to execute this task in the workplace independently, with a supervisor available on call.

### 2.3. Delphi Procedure

The Delphi technique is an iterative process, intending to obtain group consensus from different stakeholders or experts. The Delphi technique uses a multistage self-completed questionnaire with individual feedback [20]. Before we sent the questionnaire to panel members, we first tested it on five participants (not panel members), which led to small changes to the scoring system. Subsequently, we approached 120 potential panel members. The first page of the online questionnaire asked for informed consent. Subsequently, panel members were invited to rate the 46 items. They were also invited to give feedback or additional information on key and enabling competencies if they felt they needed revision or to suggest additional key and enabling competencies. In the second round, panel members were presented with the same items as in the first round and were asked to rerate or confirm their original rating of each item. They were given both their previous answers and the panel’s mean scores. Nonresponders received a maximum of two reminders. Data were stored on a protected drive, to which only the first researcher (JP) had access. Ethical approval was obtained from the Dutch Society for Medical Education (Nederlandse Vereniging voor Medische Onderwijs [NVMO]) Ethical Review Board (file no. 817).

### 2.4. Statistical Analyses

We used IBM SPSS Statistics for Windows, version 21.0 (IBM Corp., Armonk, N.Y., USA) to calculate the mean scores of each key competency and each enabling competency. Mean scores (1–6 scale) were computed across all panel members. A mean score of 4.5 or higher was rounded up to a score of 5 (a junior doctor should be able to perform this competency in the workplace under close supervision). The Delphi rounds stopped when consensus and/or stability was reached. The consensus rate was set at 75%, which was achieved when a minimum of 75% of panel members concurred that the competency belonged to one of the following categories: ‘Not applicable’ (a score of 1), ‘theoretical’ (a score of 2–3), or ‘practical’ (a score of 4, 5, or 6, all on a 1–6 scale). Stability was defined as no or minimal shifting of panel responses between rounds (one point or less on the Likert scale). The Kruskal–Wallis test was conducted to examine the differences in scores for the competencies (key and enabling) between the stakeholder groups.

## 3. Results

We approached 120 potential panel members. A total of 82 panel members responded (68%) in the first round, and 54 in the second round (66% of the respondents who had participated in the first round). Stability was achieved after two rounds, meaning that none of the scores differed more than one point on the scale between the two rounds. The results showed high levels of agreement within and between stakeholder groups: All 46 competencies need to be covered in the undergraduate curriculum. With regards to the level that competencies should be addressed the panel reached consensus on 32 of the 46 competencies (70%), but could not find consensus on 14 enabling competencies. All competencies where consensus was found were categorized as practical (score of 4, 5, or 6). Panel members concurred that graduates should be able to practice these competencies either in a simulated setting or under close supervision in a real-life setting. 

Table 2 presents the mean scores for each of the key competencies, which differed between 4.04 (standard deviation [SD] = 0.89) and 4.54 (SD = 1.01) on a six-point scale. Advance care planning received the lowest overall mean score (mean score = 4.04, SD = 0.879), meaning that the panel expected junior doctors to be able to perform this task only in a simulated setting with teacher supervision. Personal development and well-being (mean score = 4.54; SD = 1.02), communication (mean score = 4.50; SD = 0.75), and pain and symptom management (mean score = 4.48; SD = 0.74) scored highest, showing that at least a part of the panel expected junior doctors to be able to perform these tasks in the workplace under close supervision. 

Table 3 and Table S1 (Supplementary Materials) provide an overview of the mean scores for each enabling competency. The enabling competencies that panel members scored especially high were ‘communicates with respect and empathy with patients and loved ones’ (mean score = 5.02, SD = 0.96), ‘adapts to the different ways of communicating’ (mean score = 4.70, SD = 1.00), ‘adjusts content and communication style when communicating with the patient’ (mean score = 4.65, SD = 0.97), ‘determines the time and cause of death’ (mean score = 4.67, SD = 1.2), and ‘acts professionally with due regard to both personal and professional values and norms’ (mean score = 4.69, SD = 1.01). On the 14 enabling competencies, where no consensus was found regarding the level of achievement, the following enabling competencies scored lowest: ‘Integrates disease-oriented and symptom-focused care at an early stage’ (mean score = 3.38, SD = 0.91) and ‘arranges complementary care, if desired’ (mean score = 3.43, SD = 1.09). 

No significant differences were found between the panel member groups, except for the communication key competency (*p* = 0.022). Curriculum coordinators scored this key competency significantly higher than experts (*p* = 0.007) and nurses (*p* = 0.008). There was no significant difference between the curriculum coordinators, physicians, and junior doctors.

## 4. Discussion

This research has validated a competency framework for palliative care education. The panel members in this study concurred that all the proposed key and enabling competencies should be included in the undergraduate medical curricula. There was also a high level of consensus among panel members on the level of acquirement on the competencies: The panel agreed on all key and most enabling competencies that they should be attained at a practical level, i.e., in a simulated setting or in practice under close supervision. 

One significant difference between the different panel member groups was found. The curriculum coordinators and educators scored significantly higher on the communication key competency than experts in palliative care and nurses. We are not sure how to explain this, but one reason might be that curriculum coordinators felt more familiar with this subject and have more attention for teaching communication, since communication is a key domain for all area of medicine, already. Another explanation could be that curriculum coordinators find that in the communication lies the biggest educational gap. Surprisingly, we found no other differences between the different groups of panel members. We had expected, for example, that experts in palliative care would have higher expectations than the other groups.

The fact that all stakeholders agreed that junior doctors should be able to perform all key and enabling competencies on theoretical or practical level suggest that the different professions increasingly appreciate the importance of palliative care. For most competencies, participants agree on the recommended level of achievement. For some, there was no consensus on whether these should be taught only at a theoretical level or also practiced in undergraduate medical curricula.

However, implementing all this in practice will not be easy. Since teaching time and resources are limited, we foresee that it will not be feasible to incorporate all the proposed competencies into the undergraduate medical curriculum, especially if this has to happen at a practical level. Although the difference between a score of 4 and 5 may seem small, there is a large difference between teaching competencies to the level of ‘execution in a simulated environment’ (e.g., in a role play or with a simulated patient) or to the level of ‘execution under close supervision in practice’ (during internships). 

Our competency framework for palliative care can serve as a basis for integrating palliative care into existing undergraduate curricula. The question of how to implement palliative care education can have different answers. While some would argue that palliative care should be introduced as an independent course, we believe that it would be better to weave it into the existing curriculum and courses, for several reasons. First, almost all doctors will deal with terminally ill patients. Most people die in generalist settings, and physicians in nearly all specialties (rather than palliative care specialists) look after these patients in their end-of-life phase [21]. Thus, many specialties share the learning objectives of palliative care education, which, arguably, span the whole medical curriculum [22]. Research shows that palliative care education can be a vehicle to teach the patient-centered care model in undergraduate medical education and that it can contribute to personal and professional development [23]. Therefore, palliative care should be addressed in all kinds of care courses, whenever it is relevant [24]. For example, when teaching medical students about the heart, it would be logical to also familiarize them with the prospects of patients with heart failure, the potential complications and treatment options, including those aimed at quality of life in the end-of-life phase. Second, integration enhances students’ learning process because learning is contextualized. Learning about palliative care in diverse contexts is expected to enhance the transfer to practice. Vertical integration throughout the curriculum allows integration of learning tasks that gradually increase in complexity [25]. Finally, there is the practical consideration that it is difficult to free up time in the curriculum for an entirely new, independent course about palliative care. How and where competencies can be integrated in the curriculum depends on the way that the curriculum is organized, however. There is no one-size-fits-all solution. 

Our competency framework for palliative care can serve as a basis for integrating palliative care into existing undergraduate curricula. A large advantage of this framework is that, unlike previous frameworks, it incorporates the views of different stakeholders and is not solely based on the opinions of experts in palliative care. Panel members were actively involved in the study. This helped create a larger support base and a network of people motivated to increase attention to palliative care in undergraduate curricula. Our framework has further refined previous insights [7,8] and asked panel members to indicate at what level students need to acquire the proposed competencies. This has led to a framework that is concrete enough to develop education and enrich existing educational programs by integrating the palliative care context when possible. 

A limitation of this study is that it was conducted in the specific context of Dutch medical education. Fortunately, however, all Dutch medical universities were represented and the participating panel members were spread geographically throughout the country. We did not invite patients nor their family members to participate in this study. However, in an earlier conducted study, we interviewed patients who received palliative care and their family members about the palliative care that they received and on the qualities that junior doctors and nurses need to acquire. A strong point of this study is that we involved different groups of stakeholders. However, our data give only limited insight into panel members’ reasoning. We do not know why they considered the different competencies important, nor can we say anything about what they considered more urgent or important. Further research is required to investigate the usefulness of this framework in practice, and how and in which order competencies can be best addressed in undergraduate medical curricula. A natural continuation of our study would be to focus on the implementation and evaluation of the way in which these competencies are addressed and mastered by students.

## 5. Conclusions

This study has resulted in a validated competency framework that guides implementation of education about palliative care in undergraduate medical education. The level of consensus among panel members on the level of acquirement on the competencies was high. They concurred that all the proposed key and enabling competencies should be included in the undergraduate medical curricula and that all key and most enabling competencies should be attained at a practical level, i.e., in a simulated setting or practice under close supervision. The framework can be used to develop educational materials and improve medical education programs that prepare students to provide palliative care.

## Figures and Tables

**Table 1:** The five groups of participating stakeholders.

Group 1: *Round 1 N* = 21 *Round 2 N = 13*	Group 2: *Round 1 N = 21* *Round 2 N = 13*	Group 3: *Round 1 N = 13* *Round 2 N = 9*	Group 4: *Round 1 N = 13* *Round 2 N = 10*	Group 5: *Round 1 N = 14* *Round 2 N = 9*
Experts (physicians or nurses) in palliative care Who completed specific (Dutch) post-initial palliative care courses.	Physicians with specialties other than palliative care, and were tasked with educating junior doctors	Nurses with specialties other than palliative care, and who worked closely with junior doctors.	Curriculum coordinators and educators from medical universities in the Netherlands involved in undergraduate medical curricula, with at least three years’ experience in teaching.	Junior doctors who had recently graduated from medical school and were working as a junior doctor for a maximum of two years.

**Table 2:** Panel members’ mean ratings of the six key competencies in palliative care and their standard deviations (*N* panel members = 54) (1–6 scale *).

Key Competency	Junior Doctor is Able to:	Mean	Standard Deviation [SD]
Communication	Discuss the incurable illness, prognosis, and death with the patient and loved ones.	4.50	0.75
Advance care planning	Organize advance care planning in regular consultation with the patient, family, and the care providers involved.	4.04	0.89
Pain and symptom management	Combat the suffering of patients requiring palliative care and their loved ones with consideration for all four dimensions.	4.48	0.74
Working in a multidisciplinary team	Work in a multidisciplinary and interdisciplinary team of various care professionals, volunteers, and caregivers.	4.41	0.77
End-of-life care	Carry out the care trajectory around the patient’s death together with the team of professionals, volunteers, and relatives.	4.20	0.92
Personal development and well-being	Ensure personal well-being and development.	4.54	1.02

* 1: Not applicable: A newly graduated junior doctor does not require this competency; 2: The newly graduated junior doctor must possess the basic knowledge, skills, and attitudes (professional behaviour) needed for this task; 3: The newly graduated junior doctor must be able to integrate the knowledge, skills, and attitudes (professional behaviour) needed for this task; 4: The newly graduated junior doctor must be able to execute this task in an educational or simulated setting (under the teacher’s supervision); 5: The newly graduated junior doctor must be able to execute this task in the workplace under close supervision; 6: The newly graduated junior doctor must be able to execute this task in the workplace independently, with a supervisor available on call.

**Table 3:** Panel members’ highest mean ratings of the enabling competencies in palliative care and their standard deviations per key competency (*N* panel members = 54) (1–6 scale*).

Key Competency	Enabling Competency	Mean	SD
	Junior doctor is able to:		
Communication	Communicate with respect and empathy with patients and loved ones.	5.02	0.961
	Adapt to the different ways of communicating.	4.70	1.002
Advance Care planning	Explicitly discuss the patient’s wishes for the end-of-life (including euthanasia and treatment limitations).	4.43	0.716
		
	(With regard to diagnosis and treatment) take into account both the quantity and quality of life (e.g., avoids under- and over-diagnostics and weighs up diagnostic processes).	4.37	0.681
Pain and symptom management	Recognize and consider the feelings of patients and relatives and the influence these have on the well-being of those involved.	4.63	0.958
	Systematically identify the most common symptoms in the palliative phase, for example pain, respiratory symptoms, confusion, nausea and vomiting, anxiety and itching, and treat these with and without medication.	4.26	0.894
Working in a multidisciplinary team	Take advantage of opportunities for consultation in palliative care and, to this end, consult experts within and outside the institution.	4.35	0.828
	Work in a multidisciplinary and interdisciplinary team; exhibit familiarity with the duties and responsibilities of the other health care professionals involved.	4.26	0.805
End-of-life care	Determine the time and cause of death and fill in the death certificate.	4.67	1.213
	Guide the loved ones directly in the period around the death.	4.56	0.965
Personal development and well-being	Act professionally with due regard to both personal and professional values and norms.	4.69	1.006
	Exhibit knowledge of their personal responsibility as a health care professional and the limits thereof.	4.57	0.983

## Supplementary Materials

The following are available online at https://www.mdpi.com/1660-4601/17/7/2396/s1. Table S1: Panel members’ mean ratings of each enabling competency in palliative care and their standard deviations, divided into six categories (*N* panel members = 54) (1–6 scale; 1: Graduate does not require this competency; 6: Graduate must be able to practice this competency with little guidance).
