# Supplementary material for: A National, Palliative Care Competency Framework for Undergraduate Medical Curricula
Source: Int J Environ Res Public Health. 2020 Apr 1;17(7):2396. doi: 10.3390/ijerph17072396 (PMC7177526; doi:10.3390/ijerph17072396)
Supplement: Supplementary file 1 [file ijerph-17-02396-s001.pdf]

Supplementary Materials

Table S1. Panel Members' Mean Ratings of Each Enabling Competency in Palliative Care and their Standard Deviations, Divided into Six Categories (*N* panel members = 54) (1–6 scale; 1= graduate does not require this competency; 6= graduate must be able to practise this competency with little guidance).

Key Competency	Enabling Competency	Mean	SD
	Junior doctor is able to:		
Communication			
	Mark the palliative phase.	4.33	0.824
	Communicate with respect and empathy with patients and loved ones.	5.02	0.961
	Adapt to the different ways of communicating.	4.70	1.002
	Take diversity into consideration and adjust the content and communication style (e.g. with regard to cultural aspects or beliefs) when communicating with the patient.	4.65	0.974
	Keep patients and loved ones informed about the expected course, treatment and care options.	4.39	0.712
	Discuss the expectations and wishes of the patient and their relatives with regard to the course of the illness, treatment, care process and manner of death.	4.52	0.746
	Make the four dimensions of palliative care open to discussion with the patient and loved ones.	4.13	0.674
Advance care planning			
	Integrate disease-oriented and symptom-focused care at an early stage*.	3.38	0.906
	Commit to shared decision-making in setting policy with regard to the course of the illness, treatment, care process and manner of death.	4.13	0.674
	Act proactively at the various stages of palliative care and anticipate potential complications*.	3.81	0.779
	(With regard to diagnosis and treatment) take into account both the quantity and quality of life (e.g. avoids under- and over-diagnostics and weighs up diagnostic processes).	4.37	0.681
	Apply evidence-based principles from the literature, protocols and guidelines from the field of palliative care when it comes to making their own clinical decisions.	4.30	0.833
	Explicitly discuss the patient's wishes for the end of life (including euthanasia and treatment limitations).	4.43	0.716
	Act in accordance with legislation and regulations when making decisions to do with the end of life: stopping fluid/nutrition, palliative sedation, euthanasia and assisted suicide*.	3.72	0.920
	Exhibit awareness of the ethical and moral dilemmas surrounding the end of life. When necessary, discusses these issues with the patient, loved ones and care providers involved and acts according to the outcome of these considerations*.	3.89	0.793
Pain and symptom management			

	(During the palliative phase) treat problems arising from common syndromes (e.g. heart failure, COPD, cancer and dementia).	4.17	0.818
	(When setting policy) take into account and, if necessary, treat the most common emergencies arising in the palliative phase, such as delirium, (imminent) spinal cord injury, massive (lung) bleeding, vena cava superior syndrome, ileus, retention bladder, acute dyspnoea, suffocation, exhaustion, pain, existential crisis*.	3.96	0.868
	Systematically identify the most common symptoms in the palliative phase, such as pain, congested breathing, confusion, nausea and vomiting, anxiety and itching, and treat these with and without medication.	4.26	0.894
	Recognise and consider the feelings of patients and relatives and the influence these have on the well-being of those involved.	4.63	0.958
	Systematically evaluate the psychological health of the patient, and provide treatment and support or refer the patient [to a professional].	4.11	0.793
	Recognise the different coping strategies that patients and relatives use, adapt their own behaviour and communication accordingly and call in expertise and/or professional guidance as needed*.	3.91	0.896
	Recognise the different ways of mourning, adapt their own behaviour and communication accordingly and call in expertise and/or professional guidance, if needed*.	4.07	0.949
	Signal when informal care providers get overloaded and offer support/referral, if necessary*.	3.80	0.979
	Examine the patient's social context, including financial capacity and the legal and practical issues concerning end of life, and consider its influence on the patient's palliative care. Refers the patient to extra support, if required*.	3.56	1.022
	Provide space for spiritual, existential and religious topics in conversations with patients and their loved ones and refer them to specific care on request*.	3.85	0.856
Multidisciplinary team			
	Work in a multidisciplinary and interdisciplinary team; exhibit familiarity with the duties and responsibilities of the other health care professionals involved.	4.26	0.805
	Take advantage of opportunities for consultation in palliative care and, to this end, consult experts within and outside the institution.	4.35	0.828
	Organise the care chain, taking management into account*.	3.52	1.112
	Refer the patient directly to palliative care organisations that can provide care and support to palliative patients and their relatives (e.g. patient associations, informal caregiver support centres, and network organisations for palliative care) *.	3.70	0.944
	Arrange complementary care, if desired*.	3.43	1.092
End of life			
	Treat problems arising in the terminal phase, including stopping infusions and palliative sedation.	4.19	0.779
	Determine the time and cause of death and fill in the death certificate.	4.67	1.213
	Guide the loved ones directly in the period around the death.	4.56	0.965
Personal development and well-being			

	Exhibit awareness of their own feelings and know what effect they have or can have on their own attitude and behaviour.	4.46	0.985
	Reflect on distance (objectivity) and proximity in the treatment relationship with a palliative patient.	4.30	0.903
	Exhibit knowledge of their personal responsibility as a health care professional and the limits thereof.	4.57	0.983
	Recognise signs of their own overload and, if necessary, seek adequate help.	4.54	1.004
	Reflect on their own spiritual and existential experiences around life and death*.	3.87	0.933
	Reflect on own values and norms in the field of palliative care.	4.24	0.845
	Act professionally with due regard to both personal and professional values and norms.	4.69	1.006

* No consensus (lower than 75%) found
